# *Haplophyllumermenekense* (Rutaceae), a new species from Turkey

**DOI:** 10.3897/phytokeys.111.24241

**Published:** 2018-11-22

**Authors:** Deniz Ulukuş, Osman Tugay

**Affiliations:** 1 Department of Biotechnology, Faculty of Sciences, Selçuk University, Konya, Turkey Selçuk University Konya Turkey; 2 Department of Pharmaceutical Botany, Faculty of Pharmacy, Selçuk University, Konya, Turkey Selçuk University Konya Turkey

**Keywords:** Endemic, *
Haplophyllum
*, Karaman, Rutaceae, taxonomy

## Abstract

A new species of *Haplophyllum*, *Haplophyllumermenekense* (Rutaceae) is described and illustrated in line drawing. It grows on stony slopes of Ermenek town, Karaman province, in southern Turkey. It is compared with the closely related species *H.myrtifolium*. *H.ermenekense* is distinguished from the morphologically similar *H myrtifolium* chiefly by sepal shape, petal size, capsule size, presence of capsule hair and appendage form. On the other hand, the seed coat and pollen grains surface of *H.ermenekense* and *H.myrtifolium* are demonstrated in SEM photographs. In addition to the detailed description, the illustration, distribution map, conservation status and ecology of the new species are also provided.

## Introduction

With its 69 species, *Haplophyllum* Jussieu is one of the richest genera in the Rutaceae family (Townsend 1986, Navarro et al. 2004, Soltani and Khosravi 2005, Tugay and Ulukuş 2017). The genus is widely distributed in subtropical and tropical regions of the northern hemisphere of the Old World, notably in Iran, Turkey and Central Asia (Townsend 1986).

Some authors have endeavoured to subdivide *Haplophyllum* into different sections by using morphological characters (Spach 1849, Boissier 1867, Engler 1896, Vvedensky 1949, Townsend 1986). The most comprehensive studies conducted with regard to *Haplophyllum* were published by Vvedensky (1949) and Townsend (1986). In these studies, the genus was divided into four sections by Vvedensky (1949), based on capsule opening, ovule and carpel number. In the last monographic study, Townsend (1986) divided the genus into three sections according to carpel number, capsule opening, petal colour, plant architecture, stamen form and ovary shape.

*Haplophyllum* species are perennial herbs, growing mainly on sandy soil, rocky hills, slopes, stony landscapes or steppes (Townsend 1986). Morphologically, the genus is characterised by the presence of exstipulate, cymose inflorescences with, bracts, lax to dense. Flowers have five petals and five sepals, creamy-white to bright yellow petals, ten stamens with free filaments expanded below and pubescent on the inner surface. Fruits have three to five connate carpels, five-lobed capsules which are dehiscent or indehiscent (Townsend 1986). Pollen grains are tricolporate, radially symmetrical and isopolar (Ulukuş et al. 2016). The equatorial view of pollen of *Haplophyllum* is distinctly rhomboid (Townsend 1986). Tectum ornamentation is commonly striate or striate perforate (Townsend 1986, Ulukuş et al. 2016, Tugay and Ulukuş 2017).

Turkey is one of the most important centres for *Haplophyllum* diversity with three phytogeographical regions; Euro-Siberian, Irano-Turanian and Mediterranean (Ulukuş et al. 2016). Boissier (1867), in his Flora Orientalis, recognised 15 species in Turkey. At a later date, Townsend (1967) recognised 17 taxa in the Flora of Turkey. Recently, one *Haplophyllum* species was published by Tugay and Ulukuş (2017), bringing the genus to 18 taxa. With the new species described in this paper, Turkey harbours 19 *Haplophyllum* taxa, 11 (58%) of which are endemic.

The Irano-Turanian region in SW Asia is one of the richest floristic areas of the Holarctic Kingdom. Most of its species diversity is concentrated in the Anatolian plateau, Iranian plateau and Central Asia (Zohary 1973, Manafzadeh et al. 2014).

*Haplophyllum* has mostly been studied from a morphological point of view by several authors (Jussieu 1825, Spach 1849, Boissier 1867, Engler 1896, Vvedensky 1949, Townsend 1986, Salvo et al. 2011, Ulukuş et al. 2016, Tugay and Ulukuş 2017). Only a few palynological studies have been conducted in the genus *Haplophyllum* (Townsend 1986, Perveen and Qaiser 2005, Akyol et al. 2012, Al-Eisawi and Al-Khatib 2015, Ulukuş et al. 2016, Tugay and Ulukuş 2017). There are several studies about the seeds of some species belonging to the *Haplophyllum* genus (Townsend 1986, Navarro et al. 2004, Tugay and Ulukuş 2017).

From a biogeographical standpoint, Manafzadeh et al. (2014) showed that the clade, formed by the Mediterranean species of *Haplophyllum* and Anatolian *H.telephioides*, diverged from its geographically diverse sister clade in the middle Miocene probably in the Irano-Turanian region and, from there, it quickly invaded the eastern Mediterranean region.

Ermenek, located within the boundaries of Karaman province, in the Mediterranean region of Turkey, is one of the most interesting plant diversity centres in Turkey.

The aim of this study is to describe the new species, *H.ermenekense*, found in Ermenek and to compare it with similar species, especially *H.myrtifolium* Boiss., based on evidence from (micro) morphology and palynology.

## Material and methods

Between 2011 and 2015, during the process of writing a revision of the *Haplophyllum* genus in Turkey, the authors carried out fieldwork around Ermenek and collected samples. All available specimens of *Haplophyllum* harboured in Turkish herbaria (ANK, EGE, GAZI, HUB, ISTE, KNYA), relevant Turkish collections from herbaria out of Turkey (E, K), as well as all specimens collected during recent fieldwork, were examined under dissecting microscopes. Examined specimens were checked and evaluated comprehensively by relevant literature (Boissier 1867, Vvedensky 1949, Townsend 1966, 1967, 1968, 1985, 1986). The Townsend (1967, 1986) terminology was used to describe the new species.

For palynological investigations, the pollen slides were prepared according to Wodehouse’s (1935) technique. The pollen micromorphology of *H.ermenekense* and *H.myrtifolium* were examined by using scanning electron microscopy (SEM) techniques. For SEM, pollen grains were first mounted on double-sided carbon tape affixed to aluminium stubs, covered with gold with a Hummle VII sputter coater and photographed at a magnification of 2000× to 7000× with a JEOL-5600. SEM micrographs were used to determine exine sculpturing of the pollen. For pollen morphology, Punt et al. (2007) terminology was used.

Morphometric measurements of seeds were made under a stereomicroscope (Leica S8AP0) coupled to a Leica DFC 295 digital camera. The seed length and width of (10–) 30–35 seeds per species were measured. Measurements were made using the Image Tool software. Minimum-maximum ranges, mean, standard deviations of seed length and width, as well as length/width ratio, were calculated. SEM micrographs were used to determine seed coat sculpturing of the seeds. The terminology of Stearn (1983) was adopted to describe the SEM aspects of the seed coat.

## Taxonomy

### Haplophyllum ermenekense

Taxon classificationPlantaeSapindalesRutaceae

Ulukuş & Tugay sp. nov.

urn:lsid:ipni.org:names:77192112-1

**Figure 1.**
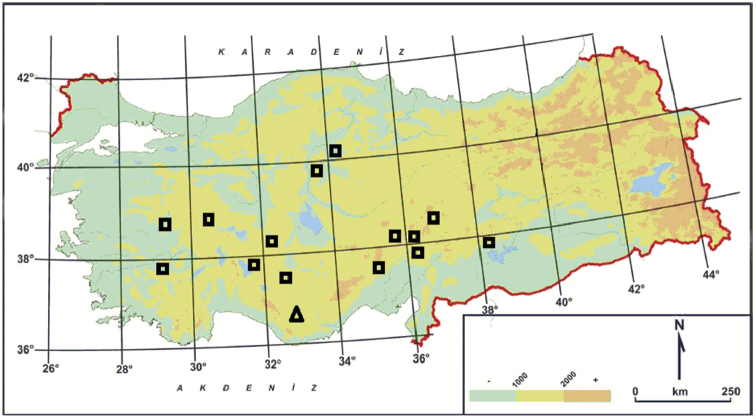
Distribution map of *Haplophyllumermenekense* (∆), *Haplophyllummyrtifolium* (□) in Turkey.

**Figure 2.**
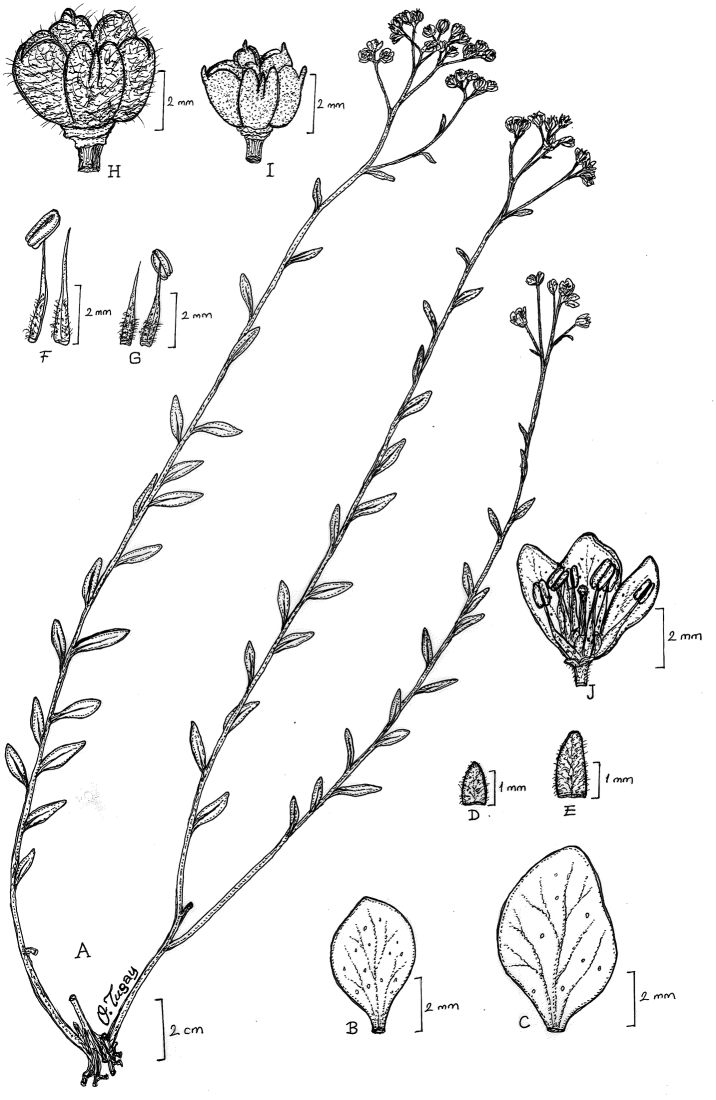
Line drawing of *Haplophyllumermenekense*. **A** habit **B** petal of *H.ermenekense***C** petal of *H.myrtifolium***D** calyx of *H.ermenekense***E** calyx of *H.myrtifolium***F** stamen of *H.myrtifolium***G** stamen *H.ermenekense***H** capsule of *H.myrtifolium***I** capsule of *H.ermenekense***J** flower of *H.ermenekense* (Drawn from the holotype by O.Tugay).

**Figure 3.**
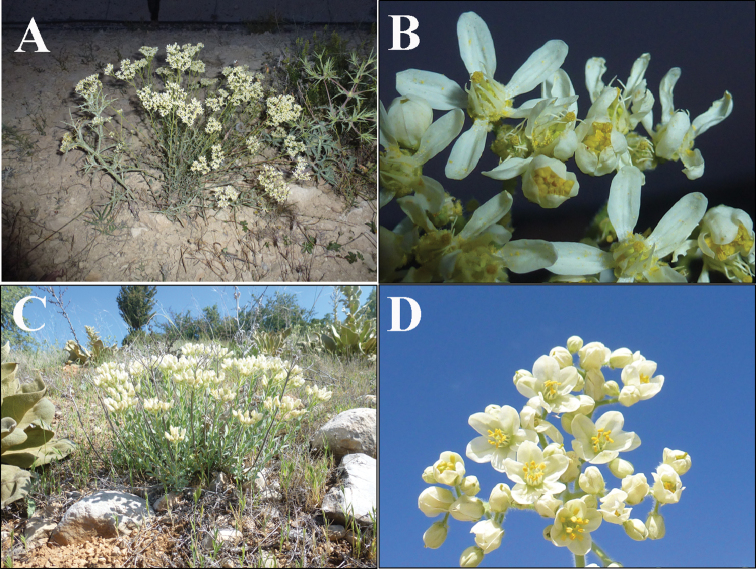
General view of habit and flowers: **A, B***H.ermenekense***C, D***H.myrtifolium*.

**Figure 4.**
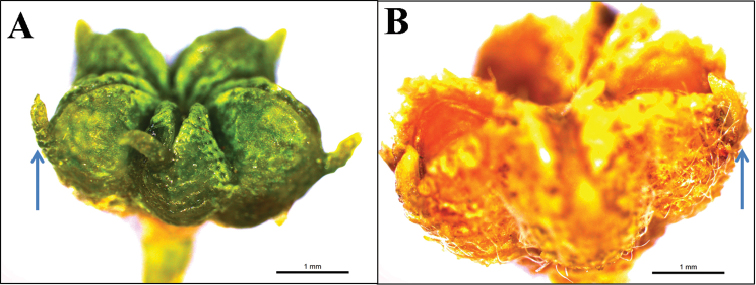
Capsules, **A***H.ermenekense***B***H.myrtifolium*.

**Figure 5.**
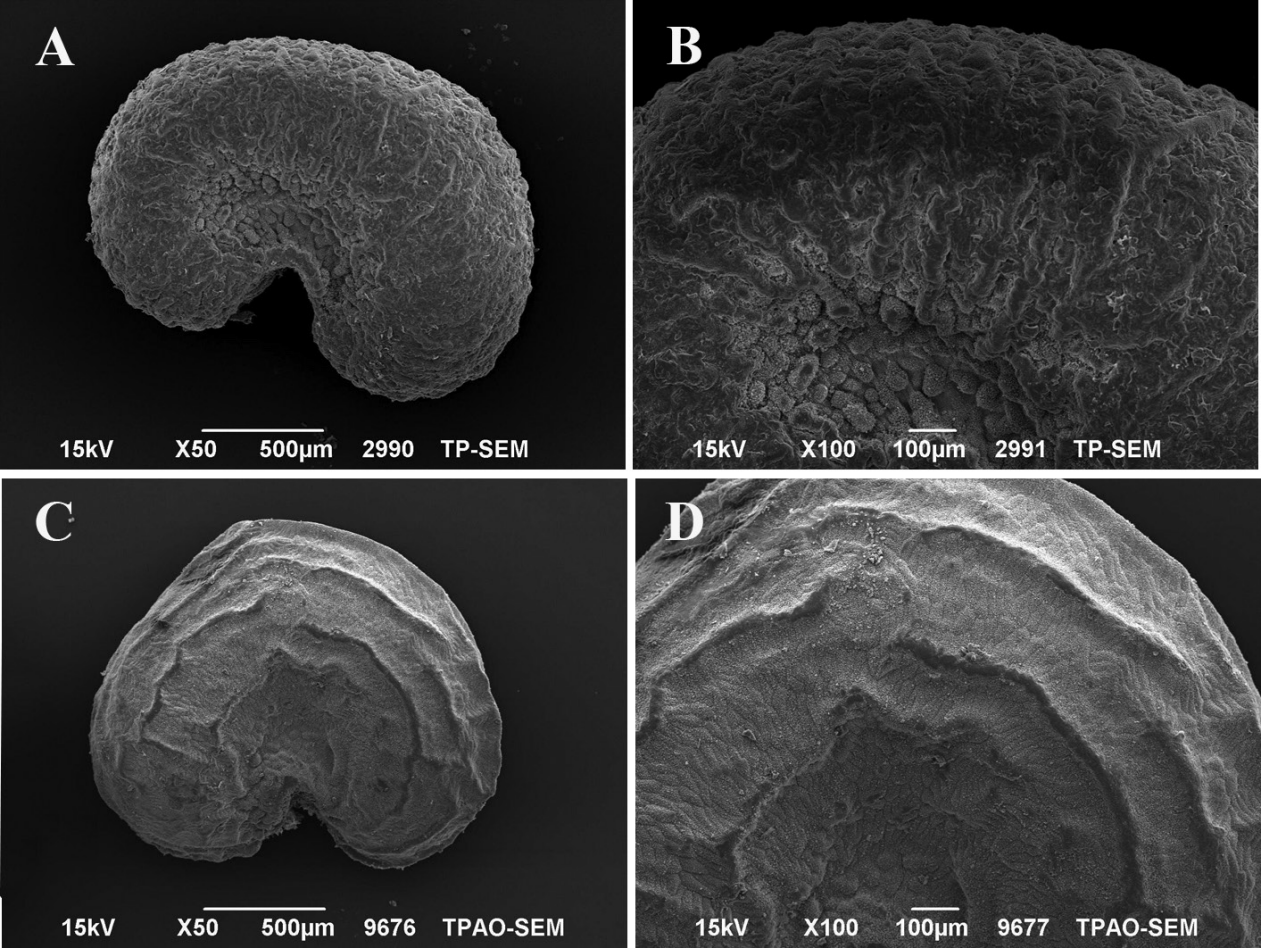
SEM photographs of seeds *Haplophyllum* species. **A, B***H.ermenekense* (*O.Tugay* 8116 & *D.Ulukuş*) **C, D***H.myrtifolium* (*O.Tugay* 8535 & *D.Ulukuş*).

**Figure 6.**
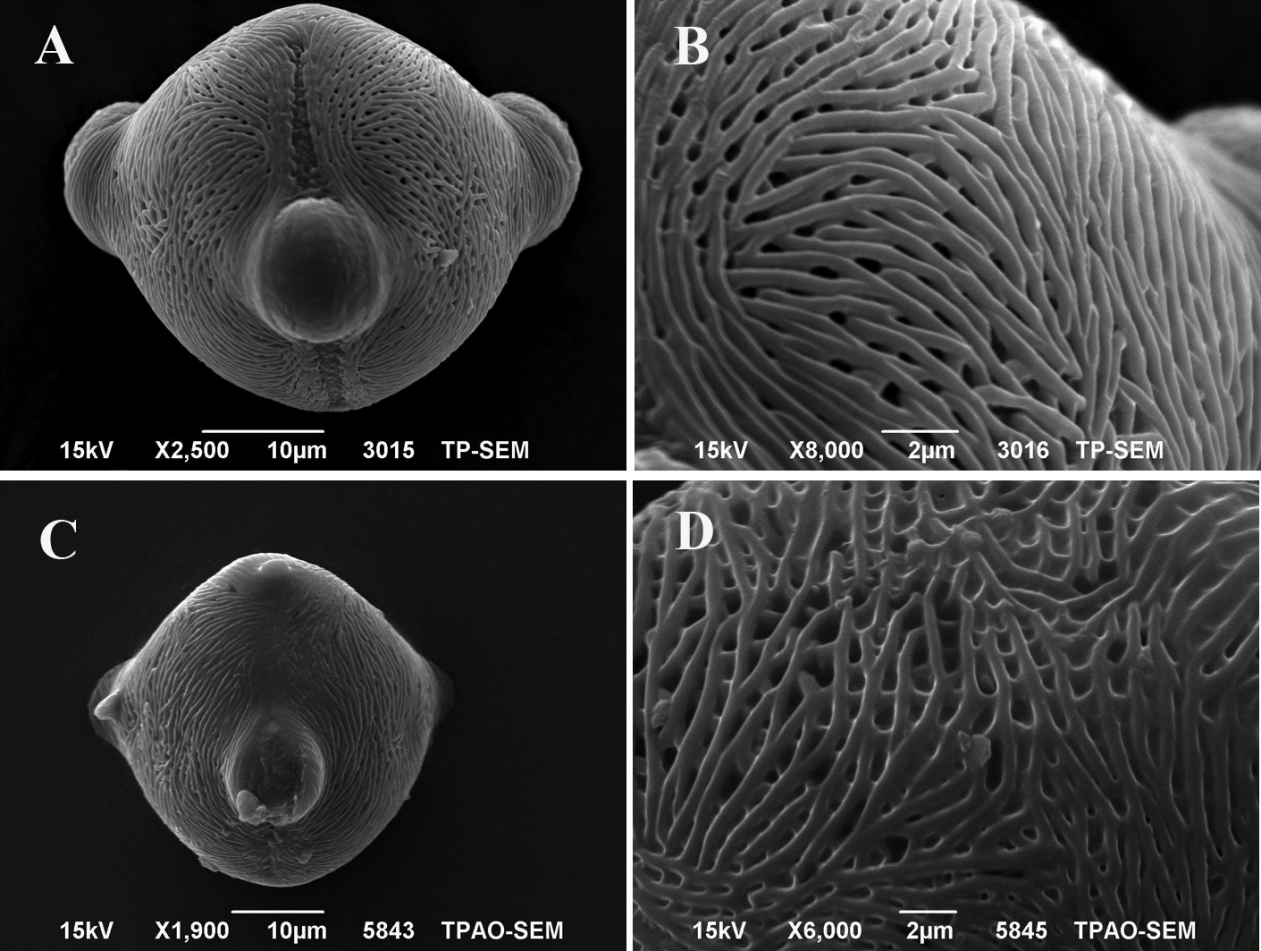
SEM micrographs of the pollen grains. **A, B** equatorial view and exine sculpturing of *H.ermenekense* (*O.Tugay* 9641 & *D.Ulukuş*) **C, D** equatorial view and exine sculpturing of *H.myrtifolium* (*D.Ulukuş 1467* & *O.Tugay*).

Figs 1 , 2 , 3 , 4 , 5 , 6

#### Diagnosis.

*Haplophyllumermenekense* most resembles the closely related *H.myrtifolium*. It differs from *H.myrtifolium* by its inflorescence usually lax form (versus dense), sepals ovate or ovate-oblong (versus lanceolate or lanceolate-oblong) and deciduous in fruit (versus persistent in fruit), petals 4–5.5 × 1.5–2.5 mm (versus 6.5–9.5 × 3.5–4.5 mm), capsule 2–2.5 × 3–4 mm (versus 3–3.5 × 5–6 mm) and glabrous (in contrast to not glabrous), with a conspicuous usually erect appendage on the outer upper surface (versus incurved appendage on the outer upper portion).

#### Type.

TURKEY. C4 Karaman; Ermenek, limestone slopes, steppe, 1200 m alt., 36°37.356'N, 32°51.543'E, 21 June 2014, *O.Tugay* 9641 & *Ulukuş* (holotype: KNYA; isotype: ANK, GAZI)

#### Description.

Perennial herbs, 25–45 cm; woody at the base with usually ascending or barely erect flowering stem with sterile shoots; stems simple below the inflorescence, furnished with rather crisped, flexuose hairs or seldom patent hairs, punctate glands. Leaves varying 8–20 × 2–8 mm, usually lanceolate or lanceolate-elliptic, both surfaces ± densely covered with flexuose-appressed to crisped white hairs, densely furnished with small, dark punctate glands; with sterile shoots present in the leaf-axils. Inflorescence lax, 4–12 cm in diameter, 10–50 flowered, the branches with flexuose hairs, with numerous punctate glands. Bracts numerous, linear-lanceolate, all ± densely white-pilose. Sepals ovate, ovate-oblong, fused at the extreme base, obtuse, white-lanate, 1–1.25 × 0.75–1 mm, with very small glands, deciduous in fruit. Petals obovate, glabrous, 4–5.5 × 1.5–2.5 mm, white, with numerous very small glands. Filaments free, narrow, somewhat expanded in the lower half, 3.5–4 mm, bearded with long hairs within about the central half, with glands very small; anthers yellow, oblong, 1.5–2 mm, Ovary segments 5, glabrous, with small acute tuberculate glands below, conical apical appendage, loculi biovulate; style glabrous, slender, 3 mm. Capsule 2–2.5 × 3–4 mm, glabrous, with a conspicuous usually erect appendage on the outer upper surface; seeds reniform, grey to black 1.25–1.5 × 1–1.15 mm, with widely spaced transverse ridges.

#### Paratypes.

TURKEY. C4 Karaman; Ermenek, Kazancı, limestone slopes, steppe, 1000 m alt., 36°28.872'N, 32°54.433'E, 21 June 2014, *O.Tugay* 9642 & *Ulukuş* (KNYA); 16 July 2012, *O.Tugay* 8116 & *Ulukuş* (KNYA); Kazancı, limestone slopes, 1200 m alt., 36°30.072'N, 32°52.433'E, 10 July 2016, *O.Tugay* 13.175 & *Ertuğrul* (KNYA).

#### Ecology.

*Haplophyllumermenekense* is endemic to Turkey. It grows at altitudes between 980 and 1200 m on limestone slopes amongst bushes (e.g. *Quercuscoccifera* L., *Juniperusoxycedrus* L., *Pistaciaterrebinthus* M.Bieb. *etc.)*. Plant diversity in this place is mainly composed of herbaceous and suffruticose plants including *Adonisflammea* Jacq., *Aegilopscylindrica* Host, *Aethionemastylosum* DC., *Capsellabursa-pastoris* (L.) Medik., *Centaureavirgata* Lam., *Digitaliscariensis* Boiss. ex Jaub. & Spach, EbenusplumosaBoiss. & Bal.subsp.speciosa Boiss.& Bal., Glauciumcorniculatum(L.)Rud.subsp.corniculatum, *Glauciumleiocarpum* Boiss., *Hyoscyamusaureus* L., *Hyoscyamusniger* L., *Isatisermenekense* Yıld., Micromeriacristata(Hampe)Griseb.subsp.cristata, *Salviaalbimaculata* Hedge & Hub-Mor. and SalviaaucheriBenthamvar.canescens Boiss. & Heldr.

#### Phenology.

Flowering time was observed at the end of July and August, mature fruits were collected in September.

#### Etymology.

The name of Ermenek town where new species found is given to the species epithet.

#### Proposed Turkish name for the new species.

Ermenek sedosu.

#### Distribution and conservation status.

*H.ermenekense* is endemic to Karaman province. It is an element belonging to the east Mediterranean phytogeographic region (Fig. 1). The range of this new species is limited to a single locality and its area of occupancy is estimated to be less than 5 km or 5 km^2^. The number of mature individual plants is estimated to be less than 250. As it is perennial, this new species has a crucial advantage for its future as destruction of the bushes by local people, road construction and deterioration of habitats may cause some threats. Thus, according to criterion D, it can be included in the EN (Endangered) category (IUCN 2001; 2016).

### Key to related *Haplophyllum* species

**Table:** 

1	Capsule without apical appendage	**2**
–	Capsule with apical appendage	**5**
2	Current year’s stem and branches and also inflorescence branches, very densely furnished with white, crisped hairs	*** H. molle ***
–	Current year’s stem and branches and also inflorescence branches, glabrous or sparsely hairy	**3**
3	Low shrub; young twigs dark purplish	*** H. amoenum ***
–	Not as above	**4**
4	Plant with numerous long, stiff, viragete stems, with the upper leaves much reduced; inflorescence small and compact, few-flowered	*** H. canaliculatum ***
–	Plant without long, stiff, virgate stems; inflorescence a broad, many-flowered corymb	*** H. viridulum ***
5	Plant vegetative parts and inflorescence branches with stipitate glandular hairs	*** H. vulcanicum ***
–	Plant not as above	**6**
6	Flowers larger, petals 10–14 mm long, upper leaves distinctly rhomboid-lanceolate	*** H. megalanthum ***
–	Flowers smaller, petals at most 9.5 mm long, leaves linear-lanceolate to eliptic-ovate	**7**
7	Capsule with a conspicous usually blunt tuberculiform appendage on the outer upper surface, leaves not exceeding 17 mm long	*** H. fruticulosum ***
–	Capsule with a conspicous usually incurved appendage or with a conspicous usually erect appendage on the outer upper surface; leaves 8–40 mm long	**8**
8	Petals 6.5–9.0 mm oblong-ovate, sepals persistent in fruit, capsule usually hairy	*** H. myrtifolium ***
–	Petals 4–5.5 mm obovate, sepals deciduous in fruit, capsule glabrous	*** H. ermenekense ***

## Seed morphology

The seed features of *H.ermenekense* and its immediate relative *H.myrtifolium* were investigated. It was seen that *H.ermenekense* has a reniform seed type. Seeds are 1.40–1.83 mm × 1.18–1.39 mm and the range of the L/W ratio is 1.28 ± 0.09. The seeds of *H.ermenekense* have widely spaced transverse ridges and micromorphologically, the sculpturing of the seed coat is not clearly striate. On the other hand, *H.myrtifolium* has narrowly spaced longitudinal ridges and a clear striate sculpturing pattern on the seed coat surface in the detailed view (Fig. 5).

## Pollen morphology

It is found that pollen grains of *H.ermenekense* are tricolporate, radially symmetrical, isopolar and their shape is oblate-spheriodal to suboblate. The measurements of pollen are as follows: polar axis (P) 40.15 ± 2.00 µm (mean ± standard deviation), equatorial axis (E) 44.44 ± 1.79 µm; the exine thickness 0.88 ± 0.11 μm and the intine thickness 0.83 ± 0.25 µm; and the ratio of P/E of pollen grains is between 0.80−0.90 µm. Exine sculpturing pattern is striate, striate-perforate or striate microreticulate (Fig. 6). Pollen features of *H.myrtifolium* have already been defined in Ulukuş et al. (2016).

## Discussion

*Haplophyllumermenekense* is morphologically similar to *H.myrtifolium*, but it differs from *H.myrtifolium* by significant vegetative and reproductive characters (Table 1).

According to Townsend (1986), *H.molle*, *H.amoenum* and *H.viridulum* have white petals yet *H.canaliculatum* has creamy-white petals. However, these species are basically separated from the other white or creamy-white flowering group without appendage. According to Townsend (1967), *H.vulcanicum* differs from *H.myrtifolium* and *H.megalanthum* by having stipitate glands on stems and inflorescence and *H.megalanthum* differs from *H.myrtifolium* by having larger corollas and rhomboid-lanceolate upper leaves. In addition, Townsend (1967) reported that *H.megalanthum* could be a large flowered variety of the western variant of *H.myrtifolium*. Ulukuş et al (2016) reported that *H.myrtifolium* differs from *H.vulcanicum* by its white to sulphur-yellow petals (versus cream-white), conical and rod-like appendages on the ovary (versus smooth line and acute), patent and crisped hairy indumentum (versus stipitate glands) and it differs from *H.megalanthum* by its lanceolate, lanceolate-elliptic and rarely ovate upper leaves (versus rhomboid-lanceolate), crisped or patent hairy leaf indumentum (versus silky), oblong-ovate (versus lanceolate-elliptic), white to sulphur-yellow (versus creamy-white) and larger petals (in *H.myrtifolium* 6−8.5 × 3−4.5 mm, in *H.megalanthum* 7−14 × 4−5 mm), conical and rod-like appendages on the ovary (versus incurve corniculus).

**Table 1.** Morphological comparison between *H.ermenekense* and *H.myrtifolium*.

Diagnostic morphological characters	* H. ermenekense *	* H. myrtifolium *
Leaf shape	usually lanceolate or lanceolate-elliptic	linear-lanceolate or ovate
Leaf size (mm)	8–20 × 2–8	40 × 2.5–12
Inflorescence form in each stem	inflorescence usually lax	inflorescence usually dense
Sepals shape	ovate, ovate-oblong	lanceolate-oblong
Sepals size (mm)	1–1.25 × 0.75–1	1.5–3 × 0.75–1.25
Petal shape	obovate	oblong-ovate
Petals size (mm)	4–5.5 × 1.5–2.5	6.5–9.5 × 3.5–4.5
Petals colour	creamy-yellow	creamy-white
Anther shape	oblong	ovate
Filament form	expanded in the lower half	expanded in the lower third to half
Filaments length (mm)	3.5–4	4.5–5
Capsule size (mm)	2–2.5 × 3–4	3–3.5 × 5–6
Capsule appendage	with a conspicuous usually erect appendage on the outer upper surface	with a conspicuous usually incurved appendage on the outer upper surface
Seed ridges type	transverse ridges	longitudinal ridges

Furthermore, *H.ermenekense* is related to *H.fruticulosum* (Labill.) G.Don, (not distributed in Turkey) differing in its hairiness, upper leaf width, capsule appendage and floral features as follows: upper leaf width 2–8 mm (versus 2–3 mm); capsule appendage conspicuous usually erect (versus blunt tuberculiform), capsule glabrous (pubescent).

Salvo et al. (2011) assembled a morphological matrix of 27 characters for 45 *Haplophyllum* species to study the similarities and differences amongst species. According to this study, *H.canaliculatum*, *H.myrtifolium* and *H.viridulum* species are similar to *H.ermenekense* in terms of flower colour character.

*Haplophyllumermenekense* is related to *H.canaliculatum* (not distributed in Turkey), differing in its apical appendage on ovary, tuberculate glands on the ovaries (versus non-tuberculate glands) and in its linear bracts (versus broad bracts).

Townsend (1986) reported that seeds of *Haplophyllum* commonly have transverse and longitudinal ridges. Tugay and Ulukuş (2017) showed that seeds are significant characters for differentiation between related species. In addition, in this study, the micromorphological study of the seeds showed that there are clear differences between the studied species. However *H.ermenekense* does not have a striate sculpturing pattern on the seed surface and *H.myrtifolium* has a distinctly striate sculpturing pattern in detailed view. On the other hand, *H.ermenekense* has transverse ridges while *H.myrtifolium* has longitudinally ridges (Fig. 5). According to Townsend’s (1986) palynologic study on some genera of the Rutaceae, including 14 species of *Haplophyllum*, it was shown that *H.myrtifolium* has striate pollen exine sculpturing. In this study, palynological results showed that there are no clear differences between the studied species. Both species have often striate-perforate and striate-microreticulate exine sculpturing patterns (Fig. 6).

## Conclusion

With the description of this new species, the number of species within *Haplophyllum* has risen to 70. This study provides material and data to aid further research on this important genus of the Rutaceae.

## Supplementary Material

XML Treatment for Haplophyllum ermenekense

## Appendix 1

Additional examined specimens

***Haplophyllummyrtifolium***; A5 Ankara: between Polatlı-Sivrihisar, Acıkır region, 840 m alt., 11 July 1991, *Z.Aytaç & H.Duman* 3871 (GAZI!); B2 Uşak: Yaparlar Village, 900 m alt., 22 July 1993 *Ö.Seçmen* 4230 (EGE!); B3 Afyon: 5 km South of Emirdağ, Bolvadin road, Lime slopes, 1100 m alt., 13 July 1965, *M.J.E Coode & B.M.G Jones* 2338 (E!); B4 Ankara: Polatlı, Acıkır region, conserved steppe, 840–850 m alt., 06 June 1990, *Z.Aytaç* 3077 (HUB!); Konya: between Cihanbeyli-Yunak, steppe, 1000 m alt., 23 July 2000, *E.Hamzaoğlu 2511* (BOZOK!); B5 Adana: Seyhan, Feke, Bakır mountain, 2000 m alt., 30 July 1952, *Davis* 19394 (E!); B5 Adana: Saimbeyli in monte Kasbel, 15 July 1893, *Manissadjian 851* (K!); C2 Denizli: Pamukkale region, 320 m alt., 19 July 1973, *O.Tugay 9638 & D.Ulukuş* (KNYA); C3 Konya: Beyşehir, Sarıköy, steppe, 1180 m alt., 16 September 2012; C4 Konya: Çumra, Apasaraycık Village, steppe slopes, 1180 m alt., 01 June 2012, *D.Ulukuş 1467 & O.Tugay* (KNYA); 28 July 2013, *O.Tugay 8535 & D.Ulukuş* (KNYA); İçel: Gülnar, Kayrak Village, 1100 m alt., 28 July 1993, *Z.Aytaç 6033* (GAZI!); C5 Adana: In monte Tauro, *Aucher-Eloy 812* (isotip, K!); C6 Kahramanmaraş: Engizek Mountain, Sırasöğüt Plateau environment, stony area, 2100–2200 m alt., 31 May 1988, *H. Duman 3948* (GAZI!); C7 Malatya: Sürgü, around Sürgü Dam, steppe, 1322 m alt., 01 July 2011, *B.Özüdoğru* 3063 (HUB!).
